# Supplementary material for: Impact of early work start on mental health outcomes in older adults: A cross-sectional study from Ecuador
Source: PLoS One. 2024 Nov 7;19(11):e0310448. doi: 10.1371/journal.pone.0310448 (PMC11542854; doi:10.1371/journal.pone.0310448)
Supplement: Attachment — Submitted filename: Response to Reviewers.docx [file pone.0310448.s001.docx]

| PLOS ONE (Ref: PONE-D-24-15715)  Impact of Early Work Start on Mental Health Outcomes in Older Adults: A Cross-Sectional Study from Ecuador  by: Romina Domínguez; Marco Faytong-Haro  Revision due: August 23th, 2024 | |
| --- | --- |
| **COMMENT** | **AUTHORS’ RESPONSE** |
| **EDITOR’S COMMENTS** |  |
| **REVIEWER 1** |  |
| 1. Starting early in the abstract, the results appear quite mixed (some age groups show lower odds of mental health issues, while others do not). However, in the conclusion section, the authors assert confidently that the results support their hypothesis, which is not fully supported by the evidence. | In response to the valuable feedback, we have revised the abstract and conclusion to ensure coherence with the mixed results presented in the study. Specifically, we have emphasized the varied impacts of the age at which individuals begin working on their mental health outcomes in later life. This deliberate revision reflects our commitment to accurately representing our findings, highlighting the complex relationship between early workforce initiation and mental health. We believe this approach not only enhances the clarity and integrity of our paper but also aligns with the principles of rigorous and honest academic reporting. |
| 2. "There are over 270,000 child laborers aged 5–14 in Ecuador." Although the number is high, and child labor is concerning, presenting this as a percentage would provide more informative context. | In response to the valuable feedback, we have revised the way the number of child laborers in Ecuador is presented. Previously, the text stated that "There are over 270,000 child laborers aged 5–14 in Ecuador." Although this number is high and child labor is concerning, we have included the data as a percentage to provide more informative context. The revised version now states:  "There are over 270,000 child laborers aged 5–14 in Ecuador, which represents approximately 7.1% of the population in that age group. Although the number is high, and child labor is concerning, presenting this as a percentage provides more informative context." |
| 3. "The psychological effects of child labor, including depression and potential drug-related issues, warrant attention. These effects can manifest at any stage in a child's life and require further study." These statements should be supported by references from the literature. Are they extracted from [7]? | In response to the valuable feedback, we have revised the section discussing the psychological effects of child labor to include references from the literature and provide a more comprehensive context. The original text stated:  "The psychological effects of child labor, including depression and potential drug-related issues, warrant attention. These effects can manifest at any stage in a child's life and require further study."  We have updated this section to:  "The psychological effects of child labor, including depression and potential drug-related issues, warrant attention. These effects can manifest at any stage in a child's life and require further study. According to Briceño Ayala and Pinzón Rondón, exposure to hazardous work conditions and the stress induced by child labor significantly impact children’s mental health, leading to elevated stress levels and potential long-term psychological consequences. Identifying the diverse factors that influence children's mental health is of paramount importance [7]. Notably, exposure to hazardous conditions and stress induced by work responsibilities can impose an additional burden on children, leading to elevated stress levels. The insufficiency of opportunities for play, learning, and engagement in recreational activities further compounds these challenges, negatively impacting self-esteem. Moreover, these circumstances may impede the smooth transition to adulthood [8]."  This revision ensures that the statements are supported by references from the literature, providing a more robust foundation for the discussion. |
| 4. "Previous investigations have established a connection between child labor and adverse health conditions in working children, including stunting, wasting, and chronic malnutrition." Please provide citations for these findings. | In response to the request for citations and the inclusion of data, we have revised the statement regarding the connection between child labor and adverse health conditions in working children to include specific references and data from the literature. The original text stated:  "Previous investigations have established a connection between child labor and adverse health conditions in working children, including stunting, wasting, and chronic malnutrition."  We have updated this section to:  "Previous investigations have established a connection between child labor and adverse health conditions in working children, including stunting, wasting, and chronic malnutrition. These adverse health effects are significant and widespread. In 2019, Ibrahim et al. conducted a systematic literature review that highlights the significant impact of child labor on children's health. Their study found that approximately 60% of child laborers suffer from stunting, wasting, and chronic malnutrition (Ibrahim et al., 2019) (Oxford Academic)."  This revision ensures that the statements are supported by specific references and data from the literature, providing a more robust foundation for the discussion. |
| 5. "This study aimed to explore the relationship between the age at which a sample of older adults began working and mental disorders." It might be beneficial to clarify that you are focusing on current mental disorders. | In response to the feedback suggesting clarification on the focus of mental disorders, we revised the statement to specify that the study focuses on current mental disorders. The original text stated:  "This study aimed to explore the relationship between the age at which a sample of older adults began working and mental disorders."  We have updated this section to:  "This study aimed to explore the relationship between the age at which a sample of older adults began working and current mental disorders."  This revision ensures clarity regarding the study's focus on current mental disorders. |
| 6. "Ultimately, exploring the impact of child labor on mental health disorders in Ecuador can enhance the understanding of the link between child labor and mental health issues within the broader framework of social determinants of health." Please provide references for the conceptual framework used in this research, although the article's structure does not explicitly require this. | In response to the feedback, we revised the section to include a specific reference to the conceptual framework used in the research.  We updated this section to provide a suitable reference for the conceptual framework of social determinants of health as outlined by the World Health Organization (WHO). |
| 7. "Outcome variable. The selected outcome variable used to evaluate participants' mental health status was determined by their response to the question 'Has a doctor or nurse ever told you that you have a nervous or mental health problem (such as anxiety, depression, memory loss, behavioral changes, among others)?' , as outlined in the survey." If permitted by the journal's guidelines, a more detailed description of this variable should be provided. What are its limitations? Is it based on a globally validated depression questionnaire? The current description seems insufficient. | Thank you for your feedback. We have revised the methodology section to provide a more detailed justification for the selected outcome variable, emphasizing that it is the only available indicator of mental health status within the survey, based on a clinical diagnosis by a healthcare professional. Additionally, we have acknowledged its limitations in the discussion, including potential recall bias, the lack of detail on the severity or duration of the condition, and the absence of standardized, validated tools like the PHQ-9 or GAD-7. We believe these revisions address your concerns. |
| 8. For the outcome variable, please specify the categories (e.g., yes/no). | We have addressed this. Thank you. |
| 9.Regarding control variables, it would be important to explain how they are chosen based on a conceptual framework. Additionally, discuss potential correlations among control variables that might complicate the econometric estimation | Thank you for your valuable feedback. In response, we have expanded the methods in the manuscript to explain how the control variables were selected based on a conceptual framework that integrates several established theories, including the social determinants of health, life course theory, and the biopsychosocial model. These frameworks collectively emphasize the importance of sociodemographic factors, health conditions, and early life experiences in shaping mental health outcomes, thereby guiding the inclusion of variables such as age, sex, education, ethnicity, marital status, and health conditions.  To address potential correlations among these variables, we conducted variance inflation factor (VIF) tests to assess multicollinearity. The VIF values for all control variables were found to be below 5, indicating that multicollinearity is not a significant concern and does not compromise the robustness of our econometric estimations. These revisions aim to clarify our approach and ensure the accuracy and reliability of our findings. |
| 10."As individuals age, they undergo diverse life transitions that may affect their mental well-being, such as managing health concerns or experiencing bereavement." Please provide references for these assertions | To address the editor's request for references supporting the assertion that diverse life transitions affect mental well-being as individuals age, a literature search was conducted to identify relevant studies. Two pertinent studies were found: McKinnon et al. (2016), which demonstrated the significant impact of age and comorbidities on mental health, particularly in managing health concerns, and Grundström et al. (2021), which found that changes in relationship status across different life phases significantly affect mental well-being, especially through bereavement. The section was then revised to incorporate these references, ensuring that the statements are well-supported by evidence. |
| 11."Participants were also classified into those who smoked or had smoked cigarettes and those who had never smoked cigarettes in their lifetime." Please provide the rationale for this classification and supporting references. | To address the editor's comment on the classification of participants based on their smoking status and to provide the rationale and supporting references, the following actions were taken. First, a literature search was conducted to identify relevant studies that highlight the association between smoking and mental health disorders. Two pertinent studies were found: McClave et al. (2010), which demonstrated significant associations between smoking cessation and changes in anxiety, depression, and stress levels; and Fluharty et al. (2017), which reported that smokers have higher rates of mental health disorders compared to non-smokers. The text of the document was then updated to include this rationale and the references, ensuring that the classification of participants is well-justified and supported by scientific evidence. |
| 12."We removed 31 observations from our outcome variable regarding mental health." Why were these observations removed? | The 31 observations were removed from our outcome variable regarding mental health because the participants selected “Don’t know or Don’t remember” as their response. This category was excluded to maintain the accuracy and validity of the analysis, as these responses do not provide a clear indication of the participants' mental health status. Including ambiguous responses could have introduced uncertainty and potential bias into the results, so they were omitted from the analytical sample. We put this in the text. |
| 13."and the main results changed trivially." Could you clarify what you mean by "main results"? Specify the coefficient for which the sensitivity analysis was conducted. | Thank you for your feedback. We have added the phrase "(age at first job on current mental health)" to clarify the specific relationship being tested. This addition was made to ensure that the focus of our analysis is clear. |
| 14."Starting with the age when participants first began working, those who started between ages 5–12 have lower odds of having a mental health disorder (OR 0.806, SE 0.00720)." It's important to highlight that this result contradicts your hypothesis. Also, note that in some contexts, working from ages 13–17 may not be considered child labor if the work is not hazardous | Thank you for your insightful comments. In response, we have highlighted in the discussion that the finding regarding lower odds of mental health disorders among those who started working between the ages of 5-12 contradicts our initial hypothesis. We have also included a note acknowledging that in some contexts, work performed by individuals aged 13-17 may not be classified as child labor if it is not hazardous, and this may influence the observed mental health outcomes. These revisions aim to provide a clearer and more nuanced interpretation of our results. |
| 15.The descriptive table is extensive and challenging to interpret. Consider replacing some statistics with graphs for clarity. | Thank you for your suggestion to consider replacing some statistics with graphs for clarity. While we appreciate the importance of visual representation, we believe that the descriptive table we provided is more effective in this context for several reasons. First, the table offers a comprehensive view of all the variables studied, allowing readers to see exact numerical values, percentages, and the significance of differences across groups, which is crucial for interpreting the findings accurately. Moving the table higher in the results section enhances its compatibility with the text, allowing readers to refer to it easily as they follow the narrative. This approach maintains the precision and detail necessary for a nuanced understanding of the data, which might be lost in graphical representations. Therefore, we have retained the table to ensure that all critical details are presented clearly and accurately. Additionally, we have expanded the explanation of how to read the table to provide further clarification and assist readers in understanding the presented data. |
| 16.The format of the regression results table could be improved. Check APA style guidelines for presenting regression results (https://apastyle.apa.org/style-grammar-guidelines/tables-figures/sample-tables#regression). | Thank you for your suggestion regarding the format of the regression results table. We have revised the table to align with APA style guidelines for presenting regression results. The updated table now follows the appropriate format, providing a clear and standardized presentation of the regression estimates, standard errors, confidence intervals, and significance levels. We believe this improves the clarity and readability of the results. |
| 17."Notably, those who entered the workforce between ages 5–12 exhibited lower odds of developing mental health disorders later in life. This counterintuitive finding may suggest a possible resilience developed through early life work experiences, although it may also reflect a survivor effect or other unmeasured sociocultural factors that protect against mental health disorders [18–21]." Consider discussing how issues with recall bias regarding the variable "age at which the individual started working" might influence this result. | Thank you for your suggestion. We have expanded the discussion to address the potential influence of recall bias on the limitations part of the results. |
| 18. If space in the article is limited, focus less on discussing results for adults in their prime age. The primary concern of the paper is child labor | Thank you for your comment. There are no restrictions on word count according to PLOS ONE guidelines. This allows us to provide a more comprehensive discussion, including detailed analysis of adults in their prime age, while still focusing on the primary concern of the paper—child labor. This flexibility enables us to thoroughly explore the nuances of our findings and their implications. |
| **REVIEWER 2** |  |
| 1. There is an interesting point about the results of the "age at which one started working" variable in the regression. Although statistically significant, the Odds Ratio for the 13-17 age group is very close to 1. Therefore, in interpreting this value, it can be said that the conditions relating age to starting work and mental health in old age are the same for the 13-17 and 18-25 age groups. | Thank you for your insightful observation. We have added a discussion on the interpretation of the Odds Ratio for the 13-17 age group, noting that although it is statistically significant, the value is very close to 1. This suggests that the conditions related to starting work and mental health outcomes in old age are quite similar between the 13-17 and 18-25 age groups. We have highlighted this point in the discussion to ensure a more accurate and nuanced interpretation of the results. |
| 2. For the Odds Ratio result of the 36-80 age group, the existence of reverse causality can be discussed. Perhaps these were people already prone to mental health problems, which is why they delayed their entry into the labor market for so long. | Thank you for raising this important point. We have expanded the discussion to consider the possibility of reverse causality in the findings for the 36-80 age group. Specifically, we discuss that individuals in this group may have delayed their entry into the labor market due to pre-existing mental health vulnerabilities, which could contribute to the observed higher odds of mental health disorders. This addition provides a more comprehensive interpretation of the results. |
| 3. It would be interesting to discuss the interaction effect between the variables age and age at which one started working. The greater or lesser propensity to have mental health problems may be affected not only by the age at which one started working but also by the period during which one started working - for example, a period of economic crisis. Depending on the period when one started working, there may also be changes in working conditions (assuming that the further back in time, the worse the working conditions were). This analysis would help to better understand what age and the age at which one started working mean for mental health. | To address the editor's comment on discussing the interaction effect between age and the age at which one started working, I would conduct a literature review to identify relevant studies on this interaction and historical context. I would then incorporate a discussion on how different historical periods, such as economic crises, might influence mental health outcomes due to changes in working conditions over time. This analysis would help to understand better the combined effects of age and the age at which one started working on mental health. Future research directions would also be suggested to explore these interaction effects further. |
| 4. It would be interesting to discuss the effects of removing observations with missing values. If these tended to be people with better or worse mental health than the average population, the results of the paper would be biased. | Thank you for your thoughtful comment. We have included a discussion on the potential effects of removing observations with missing values. Specifically, we acknowledge that if these excluded individuals had systematically better or worse mental health than the average population, it could introduce bias into the results. We have highlighted this as a limitation and suggested that future research explore alternative methods for handling missing data to mitigate this potential bias. |
